# Assessment of Palatal Masticatory Mucosa Thickness in the Saudi Population of a Teaching Hospital in the Eastern Province: A Retrospective Cross-Sectional CBCT Study

**DOI:** 10.3390/dj13070283

**Published:** 2025-06-23

**Authors:** Fatima Al Zahra, Suha Alyawar, Mohammed Alsaati, Afsheen Tabassum, Faisal E. Aljofi, Mishali AlSharief, Mohammed AlQranei, Khalid Almas

**Affiliations:** 1 Fellowship in Periodontics Program, Department of Preventive Dental Sciences, Division of Periodontics, College of Dentistry, Imam Abdulrahman Bin Faisal University, P.O. Box 1982, Dammam 31441, Saudi Arabia; 2 Department of Maxillofacial and Oral Health, King Fahd Hospital of the University, Imam Abdulrahman Bin Faisal University, P.O. Box 1982, Dammam 31441, Saudi Arabia; malsaati@iau.edu.sa; 3 Department of Periodontology, Rashid Latif Dental College/Rashid Latif Medical Complex, Lahore 54600, Pakistan; afsheen.tabassum@rlmc.edu.pk; 4 Department of Preventive Dental Sciences, Division of Periodontics, College of Dentistry, Imam Abdulrahman bin Faisal University, P.O. Box 1982, Dammam 31441, Saudi Arabia; fealjofi@iau.edu.sa (F.E.A.); msalsharief@iau.edu.sa (M.A.); msalqranei@iau.edu.sa (M.A.)

**Keywords:** palatal mucosa thickness, CBCT imaging, Saudi population, soft tissue graft, masticatory mucosa, adult population

## Abstract

**Background/Objectives:** Periodontal and implant therapies frequently require soft tissue augmentation for optimal outcomes. As the hard palate serves as the primary donor site, this study evaluated palatal masticatory mucosa thickness variations in a Saudi population of the Eastern Province using cone-beam computed tomography (CBCT) at a teaching dental hospital, providing site-specific data for clinical applications. **Methods:** A retrospective cross-sectional analysis of 215 CBCT scans from systemically healthy, non-smoking adults (>18 years) was conducted at the University Dental Hospital. Measurements were taken at 12 standardized sites (3 mm, 6 mm, and 9 mm from the cementoenamel junction) across maxillary canines, premolars, and first molars. Statistical analysis included Friedman’s test and t-tests. **Results:** Significant site variations were observed, with the second premolar region showing greatest thickness (3.48 ± 0.80 mm at 9 mm) and the first molar region the lowest (1.88 ± 0.63 mm at 3 mm) (*p* < 0.001). Mucosal thickness generally increased coronally to apically (*p* < 0.001). Age >35 years correlated with significantly thicker mucosa (*p* < 0.05), while no statistically significant gender-based differences were observed for all sites (*p* > 0.05). **Conclusions:** CBCT provides reliable, non-invasive assessment of palatal mucosa thickness. These findings offer region-specific data for consideration in periodontal and implant procedures involving soft tissue grafting.

## 1. Introduction

Periodontal and implant therapies frequently require soft tissue augmentation to ensure functional and esthetic success. The hard palate serves as the primary donor site due to its keratinized masticatory mucosa, which provides optimal properties for grafting: dense collagen fibers, rich vascularization, and adequate thickness to prevent perforation during harvest [1,2]. These characteristics make palatal mucosa ideal for both free gingival grafts and connective tissue grafts, which are critical for increasing keratinized tissue around teeth and implants, as well as for root coverage procedures [3].

The need for soft tissue augmentation is particularly evident in the Saudi population, given regional specificities in oral health epidemiology. Studies report a high prevalence of thin gingival phenotypes (47–52%) and gingival recession, especially among orthodontically treated individuals (5–47%) [4,5]. Gingival recession, defined as the apical migration of the gingival margin, poses challenges such as dentin hypersensitivity and esthetic concerns, emphasizing the importance of understanding mucosal thickness and gingival phenotype for successful treatment outcomes [6,7].

Graft survival depends heavily on revascularization, which is influenced by graft thickness and recipient site conditions. Sullivan and Atkins (1968) [8] pioneered crucial principles for successful free gingival grafts, notably establishing that graft thickness significantly impacts clinical outcomes, recommending 1.5–2.0 mm for optimal survival. Thicker grafts are easier to handle but may delay healing, while thinner grafts heal faster but are prone to shrinkage. Thus, precise evaluation of palatal mucosa thickness is essential to minimize complications and improve graft success.

Traditional methods for assessing mucosal thickness, such as bone-sounding and ultrasound, have limitations. Bone-sounding is invasive and prone to inaccuracies [1,9], while ultrasound is operator-dependent and less reliable in posterior regions [10]. Cone-beam computed tomography (CBCT) offers a superior alternative, providing non-invasive, high-resolution 3D imaging that accurately delineates soft and hard tissues [11].

This study utilizes CBCT to evaluate palatal masticatory mucosa thickness in a Saudi population, building on the refined techniques introduced by Gürlek et al. (2018) [11]. Despite numerous investigations into palatal mucosal thickness, comprehensive region-specific data for the Saudi population remain limited, particularly in comparison with other ethnic groups. This study attempts to contribute to the existing literature by providing CBCT-derived measurements of palatal tissue dimensions in this demographic. The collected data may offer helpful reference values that could assist clinicians in graft selection for periodontal and implant procedures, while potentially reducing donor-site complications. These findings, while requiring further validation, might serve as a preliminary basis for clinical decision-making in similar patient populations.

## 2. Materials and Methods

### 2.1. Sample Size

The sample size was calculated using a formula calculator. A database of 500 CBCT scans from January 2023 to December 2024 was retrieved from the Radiology Department at the University Dental Hospital, Imam Abdulrahman bin Faisal University, Dammam, Saudi Arabia. A retrospective study of CBCT scans from patients seeking dental treatment at various clinics of the IAU, University Dental Hospital, was conducted.

Assuming the mean difference in palatal masticatory mucosal thickness between men and women, µ1 = 3.66, µ2 = 3.95, σ = 0.56 [12] on 80% power, 5% margin of error, and 95% confidence interval, the minimum required sample size was at least 47 men and 47 women for gender comparison. To enable robust statistical analysis across other characteristics and subgroups (e.g., age groups, smoking status, medical history, and specific tooth types: canine, premolar, and molar), a larger sample of approximately 100 participants in each relevant group was targeted, providing sufficient power for these secondary analyses.

### 2.2. Inclusion Criteria and Exclusion Criteria

To ensure the reliability and homogeneity of the study sample, strict inclusion and exclusion criteria were applied.

#### 2.2.1. Inclusion Criteria

- Systemically healthy individuals to minimize confounding variables related to systemic conditions that might influence tissue health or healing capacity.
- Aged over 18 years.
- Not taking any medications that could potentially influence palatal mucosal thickness.
- Presence of all bilateral canine, first premolar, second premolar, and first molar teeth (these teeth demarcate the ‘safe zone’ between palatal rugae and greater palatine foramen where most grafts are harvested clinically).
- Well-aligned dental arch and normal occlusion.
- CBCT scans exhibiting sharp soft tissue contrast.
- Non-smokers, as smoking is known to negatively impact periodontal tissue health and wound healing.
- No clinical or radiographic signs of periodontal disease, including bone loss or gingival recession.

#### 2.2.2. Exclusion Criteria

- CBCT scans of poor quality (blurred or superimposed).
- Scans where the tongue was in contact with the palate during image acquisition.
- Cases presenting with missing teeth, crowding, or dental rotations.
- Individuals with palatal abnormalities such as lesions, tori, or exostosis (benign bony outgrowths), due to their potential to alter palatal tissue characteristics and confound thickness measurements.
- History of oral surgery, cancer, chemotherapy, or radiotherapy.

Patient records were systematically screened according to the predefined criteria. Only cases fulfilling all inclusion criteria without meeting any exclusion criteria were selected for CBCT analysis, yielding a final cohort of 215 scans. All demographic and health-related parameters (including medical history and smoking status) were obtained retrospectively through comprehensive electronic health records linked to each CBCT scan from the Radiology Department database.

### 2.3. Radiographic Analysis (CBCT)

All CBCT images included in the study were obtained using the same machine, a cone-beam computed tomography machine (Carestream CS 9300, Carestream Health Inc., Kodak, Rochester, NY, USA) at Imam Abdulrahman Bin Faisal University Dental Hospital. The CBCT radiographic parameters were set at 70 kVp, 10 mA, and 14.3 s scan time, according to manufacturer-recommended settings. Voxel size was 300 × 300 µm isotropic. This voxel size provides a balance between image resolution necessary for soft tissue demarcation and patient radiation dose and is considered clinically acceptable for accurate measurements in such studies. Large Field of View (16.5 cm diameter by 13 cm length) was used, following the ALARA (as low as reasonably achievable) principle. Images were reconstructed and analyzed using Carestream viewer (CS 3D Imaging v3.7.1).

**Figure 1:**
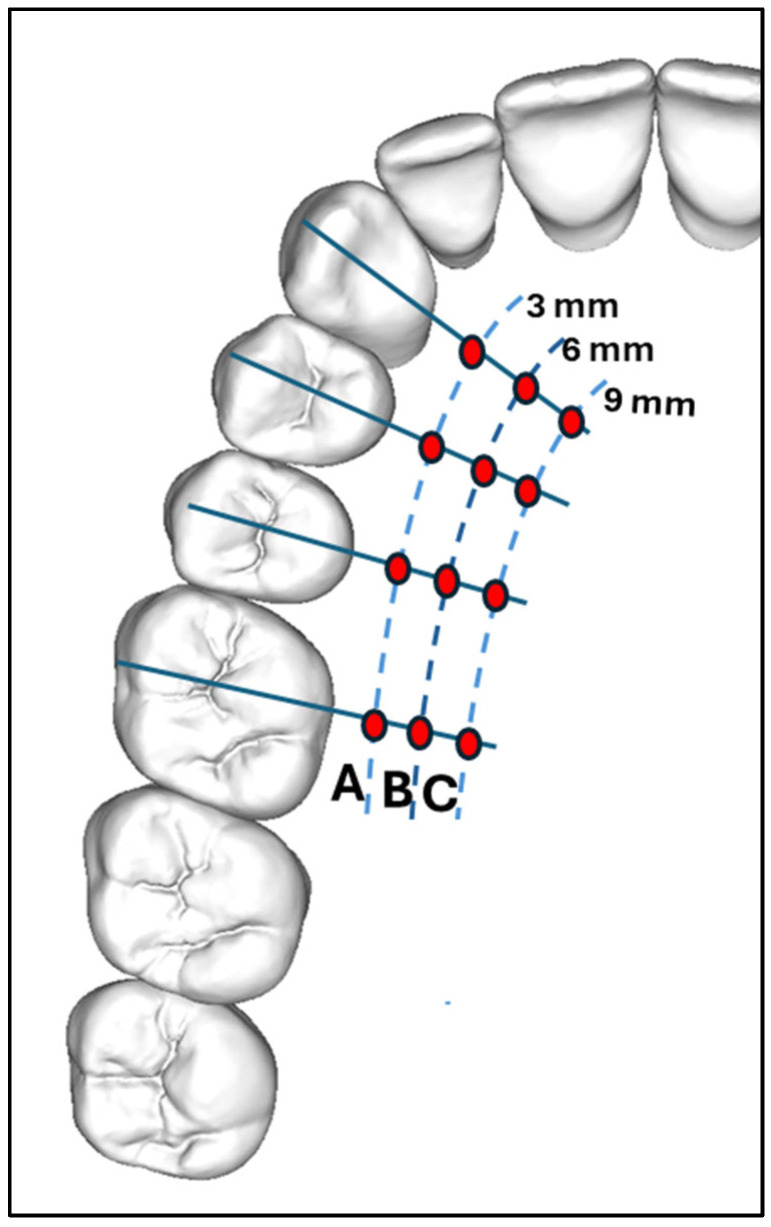
Determination of measurement position. Twelve measurement sites were defined according to the distance from the margins (A, 3 mm; B, 6 mm; and C, 9 mm) and tooth line-related positions canine, first premolar, second premolar, first molar.

**Figure 2:**
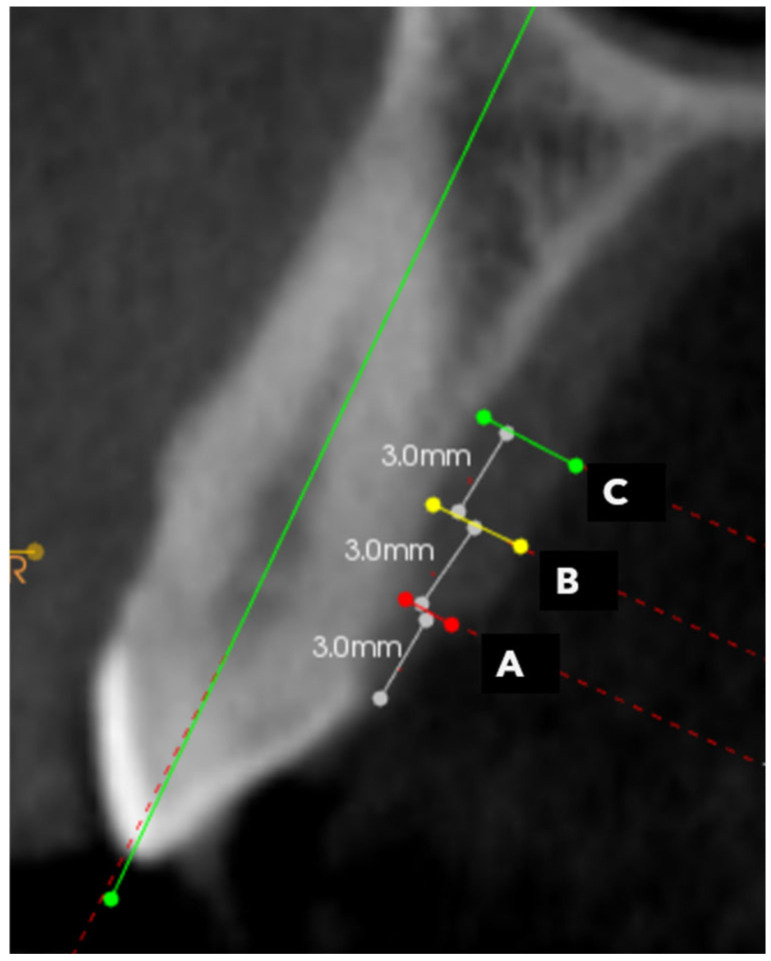
CBCT image of the measurement sites (A: 3 mm, B: 6 mm, C: 9 mm from CEJ).

Imaging analyses were viewed on a Monitor, calibrated using TG18 quality control model from the American Association of Medical Physicists (AAMP). The monitor was placed in a dimly lighted room that had no windows or ambient light exposure. Carestream software version (CS 3D Imaging v3.7.1) was used by uploading DICOM radiographs from a CBCT machine. In the CBCT analysis, each maxillary tooth—canine, first premolar, second premolar, and first molar—was evaluated unilaterally on the dentated side. In the axial view, each tooth was bisected mesiodistally to identify its precise midpoint. The sagittal view was then used to align the cursor along the long axis of the tooth, extending from the tip of the crown to the root apex. In the coronal view, palatal mucosal thickness was measured at three standardized points along the long axis—3 mm (Site A), 6 mm (Site B), and 9 mm (Site C) from the cementoenamel junction—perpendicular to the tooth axis. This resulted in a total of 12 measurement sites across the four tooth types, as illustrated in Figure 1 and Figure 2.

The thickness of the palatal soft tissue was measured at each point as the distance between the hard tissue, either root surface or alveolar bone, and the soft tissue border.

### 2.4. Inter- and Intra-Examiner Calibration

A random sample of 10 CBCT scans (5 male and 5 female participants) was selected to evaluate measurement reliability. To assess intra-examiner consistency, the same examiner performed measurements twice with a one-week interval between evaluations. For inter-examiner reliability, measurements were compared between the primary examiner and a blinded consultant radiologist. Statistical analysis included paired-sample t-tests to compare mean differences in mucosal thickness measurements with 95% confidence intervals, along with correlation coefficients to quantify agreement between assessors. This calibration protocol ensured measurement consistency while minimizing observer bias.

### 2.5. Statistical Analysis

Statistical analysis was carried out by using SPSS version-29.0 (IBM, Armonk, NY, USA). The qualitative variables like gender were presented as frequencies and percentages. The continuous response variables like age and palatal masticatory mucosal thickness were presented as mean ± standard deviation. Non-parametric Friedman’s test of k-related samples was applied to compare the mean thickness of three different sites each for canine, 1st premolar, 2nd premolar, and 1st molar, whereas an unpaired t-test was applied to compare mean thickness between the genders and the age groups (35 or below and above 35 years). The age cutoff of 35 years was chosen to dichotomize the adult population into two distinct groups, facilitating the analysis of age-related trends in mucosal thickness, consistent with similar demographic stratifications used in previous dental research examining tissue maturation and aging effects. A *p*-value less than or equal to 0.05 was considered statistically significant. It is important to note that no formal correction for multiple comparisons was applied to the site-by-site analyses. This approach was chosen to allow for individual exploration of specific measurement points; however, it should be considered when interpreting the overall statistical significance of multiple comparisons.

## 3. Results

### 3.1. Intra-Examiner Consistency

The Cronbach’s alpha reliability analysis demonstrated high item reliability for the measurements, with Cronbach’s alpha coefficients of α = 0.975. The intra-observer error analysis revealed no significant difference in palatal masticatory mucosal thickness measurements between assessments (*p* > 0.05).

### 3.2. Inter-Examiner Consistency:

The mean difference in the thickness of palatal masticatory mucosa on 12 sites were non-significant and had strong correlation, except 2 sites, i.e., first premolar b, and second premolar b, which were significant at *p* = 0.023 and *p* = 0.041, respectively. However, there was significantly strong correlation between the assessors for these measurements (Table 1).

### 3.3. Demographic Data

**Figure 3:**
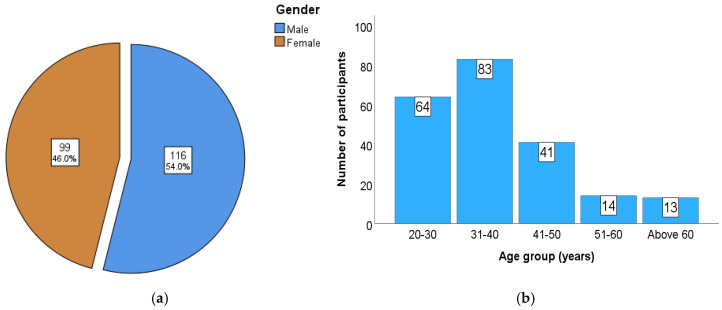
(**a**) Gender-wise distribution; (**b**) age-wise distribution.

The study included 215 participants. Figure 3a demonstrates the gender distribution of the study participants, with a slightly higher representation of male participants (54%) compared to female participants (46%). The overall mean age of the participants was 37.48 ± 11.66 years, ranging from 20 to 81 years. Figure 3b illustrates the age-wise distribution, showing that the 31–40-year age group was the most prevalent, accounting for 38.6% (n = 83) of participants. This was followed by the 20–30-year group (29.8%), 41–50-year group (19.1%), 51–60-year group (6.5%), and those above 60 years (6%). There was no statistically significant difference in the mean age between male and female participants (*p* = 0.373). with mean ages of 38.20 ± 12.1 and 36.77 ± 11.24 years, respectively.

### 3.4. Comparison of Mean Thickness

**Figure 4:**
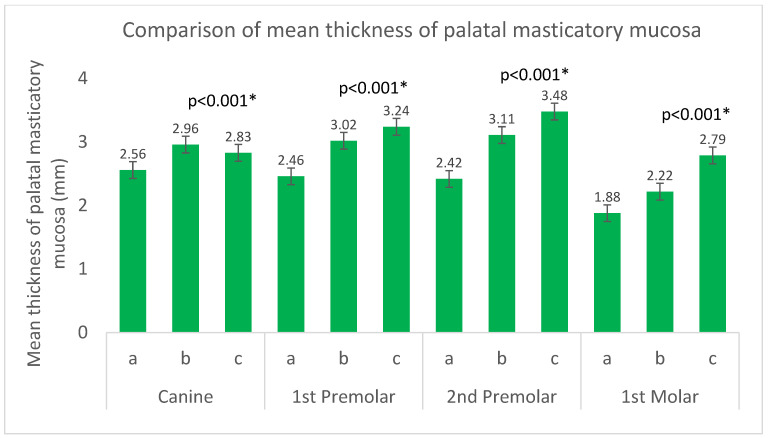
Comparison of mean thickness of palatal masticatory mucosa across sites and tooth types (Friedman’s test). Error bars represent standard deviation. * Statistically significant at (*p* < 0.001).

Table 2 presents the mean thickness and standard deviation (in millimeters) of the palatal masticatory mucosa at three specific sites (a, b, and c) for each of the four tooth types: canine, first premolar, second premolar, and first molar. The table reveals a statistically significant difference in mucosal thickness across the three sites for each tooth type (*p* < 0.001), as determined by Friedman’s test. Generally, for all tooth types, site c exhibits the greatest mean mucosal thickness, while site a has the lowest. Overall, the mean mucosal thickness increases progressively from site a to site c. Notably, the second premolar region consistently exhibited the highest mean thickness at site c, while the first molar region showed the lowest thickness at site a, as visually summarized in Figure 4.

**Figure 5:**
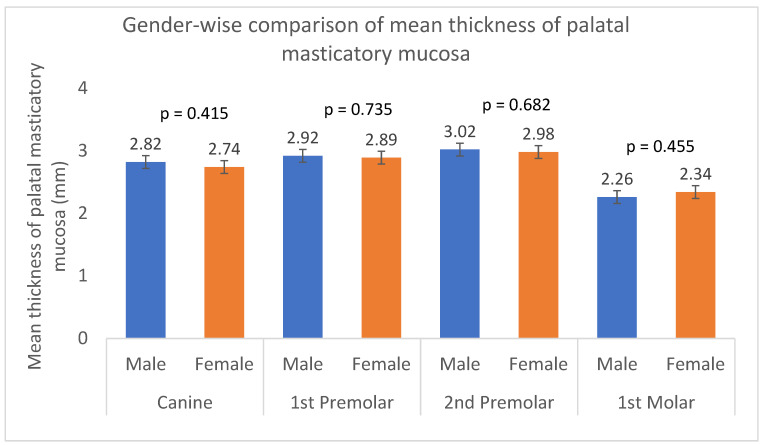
Gender-wise comparison of mean thickness of palatal masticatory mucosa (analyzed using unpaired *t*-test). Error bars represent standard deviation.

There was no statistically significant difference in the mean palatal masticatory mucosa thickness between male and female participants at any of the 12 sites (*p* > 0.05) (Table 3). (Figure 5 visually represents the mean palatal mucosal thickness at each of the 12 sites, separated by gender (male and female). The graph would likely show overlapping or very similar bar heights with their respective error bars, reflecting the lack of statistically significant differences reported in Table 2.

**Figure 6:**
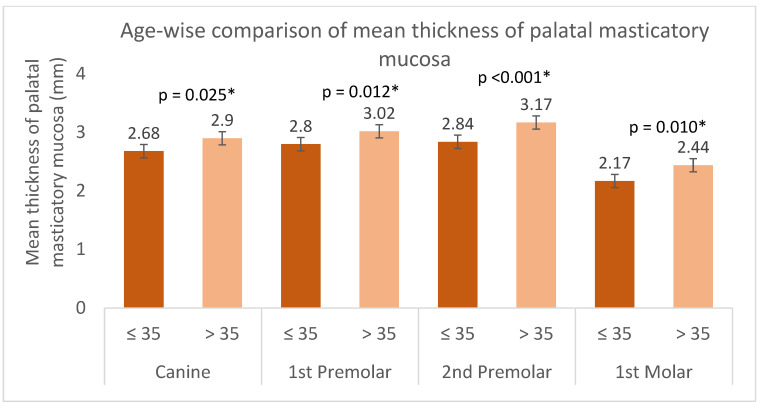
Age-wise comparison of mean thickness of palatal masticatory mucosa (analyzed using unpaired *t*-test). Error bars represent standard deviation. * Statistically significant at (*p* < 0.05).

A statistically significant difference was observed in the mean palatal masticatory mucosa thickness between the age groups ≤35 years and >35 years at a 5% significance level (Table 4). Figure 6 presents the mean palatal mucosal thickness at each of the 12 sites, separated by the two age groups (≤35 years and >35 years). Based on Table 3, this graph would likely show consistently higher bars for the >35 years age group compared to the ≤35 years age group, particularly at sites where statistical significance was noted (indicated by ‘*’ in Table 4).

## 4. Discussion

This study aimed to evaluate the mean thickness of the palatal masticatory mucosa in a Saudi population using CBCT imaging. The results provide valuable data on the palatal mucosal thickness at specific sites, which is crucial for the strategic planning and skillful implementation of various periodontal procedures, including soft tissue grafting. While each patient presents unique anatomical characteristics, the normative data and site-specific trends identified in this study offer a foundational guide for clinicians. This information can help in predicting donor tissue availability, minimizing donor-site morbidity, and optimizing surgical outcomes by enabling a more informed selection of graft harvest sites tailored to the patient’s specific needs.

This study utilized cone-beam computed tomography (CBCT) to assess palatal masticatory mucosa thickness, a method that offers several advantages over traditional techniques. CBCT provides a non-invasive, three-dimensional visualization of the palatal tissues, allowing for accurate measurements at pre-determined sites and minimizing the potential for soft tissue compression that can occur with direct clinical measurements [13,14]. This is particularly important for surgical planning, as it provides a more realistic representation of tissue availability for grafting procedures. While some studies have employed direct clinical measurement via bone-sounding [1,15], CBCT’s ability to delineate soft tissue contours and its spatial accuracy make it a valuable tool for detailed anatomical assessment [16,17]. Moudi et al. (2019) demonstrated that CBCT accurately measures soft tissue thickness (0.1 mm accuracy, especially under 2 mm), which is clinically significant in periodontal plastic surgery and implantology [18]. However, it is important to acknowledge the limitations of CBCT, including the associated radiation dose and the potential for image artifacts, which were considered in our study design and data interpretation [9].

The high reliability of the measurements, as indicated by the Cronbach’s alpha values and the absence of significant intra- and inter-observer errors, confirms the accuracy and consistency of the data obtained in this study. This contributes to the reliability of the measurements and supports their use for clinical practice within the studied population.

The findings of this study indicate that the mean thickness of the palatal masticatory mucosa varies significantly across different sites within the palate. In the second premolar region, it showed maximum thickness (3.48 ± 0.80mm), while first molars were thinnest (1.88 ± 0.63mm), which corroborates the clinical practice of preferentially harvesting from premolar regions. Consistent with our findings, a study conducted in Turkey by Burak and Hilmi (2024) also observed that the mucosal thickness in the palatal region generally increases from anterior (mean: 1.81 mm) to posterior (mean: 3.06 mm) [19]. This comparison highlights the importance of collecting region-specific data, as while general trends may align, population-specific anatomical variations, such as those in the Saudi Arabian population investigated in our study, remain crucial for precise clinical applications. This thickness gradient explains why premolar areas allow safer harvesting of 1–2mm thick grafts without risking palatal perforation [15]. The statistically significant differences observed across sites, particularly the higher thickness in the premolar regions, directly translate to greater tissue availability and reduced risk during graft harvesting, a finding with clear clinical relevance for donor-site selection. Several studies suggest the mucosa tends to be thicker in the second premolar region with a mean thickness of 3 mm, although Müller et al. (2000) [20] also noted thicker mucosa in the third molar region [20]. In contrast, other areas such as the molar region or over the palatal root of the maxillary first molar have been reported to have thinner mucosa. Studies by Yilmaz et al. (2015) [21] on greater palatine foramen location and Tavelli et al. (2018) [2] on minimizing patient morbidity underscore the importance of detailed anatomical considerations of the palate when planning surgical procedures involving graft harvesting. These variations are clinically significant because they influence the amount of tissue available for grafting and the potential for donor-site morbidity.

While there are inconsistencies in gender-related findings, several studies converge on the observation that the premolar region is an area of relatively substantial palatal mucosa thickness. Studer et al. (1997) [1] noted that the mucosa was thickest in the canine–premolar region. Both Müller et al. (2000) [20] studies also highlighted the premolar area as having thick palatal mucosa. Wara-aswapati et al. (2001) [22] further support this, identifying the canine and premolar areas as suitable donor sites for grafting.

The impact of gender on mucosal thickness remains a debated issue; however, no significant differences were observed in palatal mucosal thickness between male and female participants in our study. This suggests that gender may not be a primary determinant of palatal mucosal thickness within the Saudi population, aligning with previous reports by Studer et al. (1997) [1] and Wara-aswapati et al. (2001) [22], who also found no statistically significant gender-related differences. However, it is important to acknowledge that other factors, such as hormonal variations or individual anatomical differences, could potentially play a role, although they were not assessed in these studies. Notably, our findings contrast with some previous reports; for instance, Müller et al. (2000) [20] observed a statistically significant thinner mucosa in women (mean 0.3 mm) compared to men in the canine to second molar region. These inconsistencies highlight that the influence of gender on palatal mucosa thickness is a subject of debate, indicating the need for further research to clarify its role and clinical implications, especially in procedures like tissue grafting.

A significant finding of this study was the effect of age on palatal mucosal thickness. The results demonstrated that older individuals (>35 years) tend to have thicker palatal mucosa compared to younger individuals (≤35 years). The observed association between increased palatal mucosa thickness and age may be attributed to various age-related changes in the connective tissue and epithelium of the oral mucosa, including changes in tissue composition, collagen density, or mucosal remodeling. When interpreting the results of this study, it is important to consider the age of the participants in comparison to other studies. Our study included individuals over 18 years of age, representing a broader adult population. In contrast, Müller et al. (2000) [20] specifically examined young adults (19–30 years), while Wara-aswapati et al. (2001) [22] directly investigated the impact of age, reporting increased palatal mucosal thickness with increasing age. Clinically, this age-related difference may have implications for graft harvesting, as older patients might provide a greater amount of donor tissue. This suggests that age could be a valuable pre-operative consideration, potentially allowing for the harvest of thicker and more robust grafts in older individuals, thereby influencing graft survival and recipient site outcomes.

This study offers several notable methodological strengths. The use of CBCT imaging enabled accurate, non-invasive three-dimensional assessment of palatal mucosal thickness, overcoming limitations associated with traditional measurement techniques. A standardized measurement protocol was rigorously implemented across all 215 scans, ensuring consistency in data collection. The relatively large sample size enhances the statistical power and reliability of our findings. Furthermore, strict inclusion/exclusion criteria helped maintain sample homogeneity while minimizing confounding variables.

Several key limitations should be considered when interpreting these results. Firstly, the cross-sectional design, while efficient for establishing baseline measurements, inherently limits our ability to evaluate longitudinal changes in mucosal thickness or establish cause-and-effect relationships. Secondly, the data originates from a single teaching hospital in one Saudi Arabian province, which introduces geographic restriction and may limit the generalizability of our findings to the broader Saudi population or other ethnic groups. The retrospective nature of the study also carries an inherent potential for selection bias, as patients were selected based on pre-existing CBCT scans rather than specific recruitment for this study. Although CBCT provides superior imaging capabilities compared to alternative methods, it carries inherent limitations including radiation exposure and potential image artifacts. The 0.3 mm voxel size, while clinically acceptable, may have introduced partial volume averaging effects that could be mitigated in future studies through higher resolution scanning (0.1–0.15 mm). Additionally, we did not account for potential modifying factors such as periodontal status, systemic conditions, tobacco use history, socioeconomic status, dental treatment history, body mass index (BMI), palatal vault morphology, or root prominence which may influence mucosal thickness measurements. As this study is specific to the Saudi population, the lack of comparative data from other ethnic groups restricts the generalizability of the findings. Nonetheless, the identified factors highlight important areas for future research to achieve a more comprehensive understanding of palatal anatomy relevant to grafting procedures.

Building on these findings, several promising research avenues emerge. Future investigations should examine the relationship between gingival phenotype classification and site-specific mucosal thickness variations. Longitudinal study designs would provide valuable insights into age-related changes in palatal mucosa over time. Multi-center collaborations could help establish population-specific normative data while controlling for geographic and ethnic variables. Additionally, clinical outcome studies correlating pre-operative CBCT thickness measurements with post-grafting tissue stability would help refine surgical protocols. Such research efforts would advance evidence-based decision-making in periodontal plastic procedures.

## 5. Conclusions

This CBCT-based study provides comprehensive data on palatal masticatory mucosa thickness in a Saudi population, revealing significant site-specific variations. The second premolar region emerged as the thickest, making it an optimal donor site for soft tissue grafting, while the first molar region was the thinnest, aiding in avoiding intra-operative complications. Age was a key determinant, with individuals >35 years exhibiting thicker mucosa, suggesting potentially more abundant donor tissue that could be considered during surgical planning to optimize outcomes, whereas gender showed no significant influence. These findings equip clinicians with evidence-based insights regarding palatal mucosal thickness distribution within the studied population, which can aid in surgical planning and potentially contribute to minimizing donor-site morbidity in periodontal therapies. However, it is crucial to note that direct clinical validation studies are needed to confirm the impact of these specific measurements on ultimate treatment outcomes. Future research should explore the role of gingival phenotype and longitudinal graft performance to further refine clinical protocols. Specifically, prospective studies comparing pre-operative CBCT measurements with intra-operative soft tissue thickness and long-term graft stability are recommended.

## Figures and Tables

**Table 1:** Inter-examiner consistency test for measurement of mean thickness of palatal masticatory mucosa in mm at the subject level.

Tooth	Site	Difference Mean ± SD	95% Confidence Interval	*p*-Value	Correlation Coefficient (r)	*p*-Value
Canine	a	−0.01 ± 0.28	−0.21–0.19	0.912	0.812	0.004
	b	0.03 ±0.25	−0.15–0.21	0.708	0.919	<0.001
	c	−0.01 ± 0.56	−0.41–0.39	0.956	0.727	0.017
1st Premolar	a	0.05 ± 0.24	−0.12–0.22	0.521	0.878	<0.001
	b	0.28 ± 0.32	0.05–0.51	0.023	0.893	<0.001
	c	0.01 ± 0.25	−0.17–0.19	0.901	0.939	<0.001
2nd Premolar	a	0.04 ± 0.22	−0.12–0.20	0.583	0.891	<0.001
	b	0.23 ± 0.31	0.01–0.45	0.041	0.908	<0.001
	c	−0.01 ± 0.23	−0.17–0.15	0.893	0.958	<0.001
1st Molar	a	0.03 ± 0.59	−0.39–0.45	0.875	0.569	0.086
	b	−0.01 ± 0.42	−0.31–0.29	0.942	0.701	0.024
	c	0.00 ± 0.64	−0.45–0.45	10.000	0.699	0.024

**Table 2:** Mean thickness of palatal masticatory mucosa in mm at the subject level.

Site	Canine	1st Premolar	2nd Premolar	1st Molar
a	2.56 ± 0.65	2.46 ± 0.55	2.42 ± 0.60	1.88 ± 0.63
b	2.96 ± 0.71	3.02 ± 0.66	3.11 ± 0.76	2.22 ± 0.77
c	2.83 ± 0.80	3.24 ± 0.72	3.48 ± 0.80	2.79 ± 0.94
Mean ± S.D	2.78 ± 0.72	2.91 ± 0.65	3.00 ± 0.72	2.30 ± 0.78
Sig.	<0.001 *	<0.001 *	<0.001 *	<0.001 *

* Friedman’s test of k-related samples revealed significant difference in mean thickness of three different sites.

**Table 3:** Mean thickness of palatal masticatory mucosa in mm by gender (analyzed using unpaired *t*-test).

	Canine			1st Premolar			2nd Premolar			1st Molar		
Site	Male (*n* = 116)	Female (*n* = 99)	Sig.	Male (*n* = 116)	Female (*n* = 99)	Sig.	Male (*n* = 116)	Female (*n* = 99)	Sig.	Male (*n* = 116)	Female (*n* = 99)	Sig.
a	2.56 ± 0.64	2.56 ± 0.65	0.995	2.43 ± 0.55	2.49 ± 0.56	0.473	2.42 ± 0.62	2.42 ± 0.57	0.992	1.91 ± 0.64	1.85 ± 0.61	0.507
b	3.03 ± 0.77	2.88 ± 0.62	0.135	3.03 ± 0.68	3.01 ± 0.64	0.767	3.12 ± 0.76	3.10 ± 0.76	0.806	2.17 ± 0.79	2.28 ± 0.75	0.289
c	2.88 ± 0.88	2.77 ± 0.71	0.330	3.30 ± 0.76	3.17 ± 0.68	0.168	3.53 ± 0.79	3.42 ± 0.81	0.300	2.71 ± 0.93	2.88 ± 0.96	0.188
Mean ± S.D	2.82 ±0.76	2.74 ± 0.66	0.415	2.92 ± 0.66	2.89 ± 0.63	0.735	3.02 ± 0.72	2.98 ± 0.71	0.682	2.26 ± 0.79	2.34 ± 0.77	0.455

**Table 4:** Mean thickness of palatal masticatory mucosa in mm by age group (analyzed using unpaired *t*-test).

	Canine			1st Premolar			2nd Premolar			1st Molar		
Site	≤35 (n = 111)	>35 (n = 104)	Sig.	≤35 (n = 111)	>35 (n = 104)	Sig.	≤35 (n = 111)	>35 (n = 104)	Sig.	≤35 (n = 111)	>35 (n = 104)	Sig.
a	2.44 ± 0.59	2.68 ± 0.68	0.006	2.41 ± 0.55	2.51 ± 0.56	0.199	2.29 ± 0.57	2.55 ± 0.60	0.002	1.83 ± 0.59	1.93 ± 0.67	0.248
b	2.86 ± 0.68	3.07 ± 0.73	0.033	2.90 ± 0.57	3.15 ± 0.72	0.007	2.91 ± 0.66	3.33 ± 0.79	<0.001	2.06 ± 0.67	2.40 ± 0.84	0.001
c	2.73 ± 0.74	2.94 ± 0.86	0.050	3.08 ± 0.66	3.41 ± 0.76	<0.001	3.33 ± 0.79	3.63 ± 0.79	0.006	2.60 ± 0.82	3.00 ± 1.03	0.002
Mean ±S.D	2.68 ± 0.67	2.90 ± 0.76 *	0.025	2.80 ± 0.59	3.02 ± 0.68 *	0.012	2.84 ± 0.67	3.17 ± 0.73 *	<0.001	2.17 ±0.69	2.44 ±0.84 *	0.010

* Statistically significant at (*p* < 0.05).

## Data Availability

All supporting data are included within the article.
